# Investigation of Stem cells Technology in The Light of Jurisprudential Documents 

**DOI:** 10.22074/cellj.2020.5694

**Published:** 2019-09-08

**Authors:** Ahmad Pourebrahim, Iraj Goldouzian, Ahmad Ramezani

**Affiliations:** 1 Department of Law, Qeshm Branch, Islamic Azad University, Qeshm, Iran; 2 Department of Law, Tehran University, Tehran, Iran; 3 Department of Law, University of Knowledge and Culture, Tehran, Iran

**Keywords:** Embryo, Jurisprudential, Law, Stem Cell

## Abstract

**Objective:**

The aim of this study is investigation of Stem cells Technology in The Light of Jurisprudential Documents.

**Materials and Methods:**

In this analytical-descriptive research, we collected the relevant data through a literature search. We have used PubMed, ScienceDirect, Google Scholar, Iranian databases like SID, Iran doc, Iranian law and also Islamic resources for this study.

**Results:**

There are so many controversies about safety of these cells and possible dangers for human body. As in Iran, laws of stem cells are not clear. Elimination of barriers requires drafting laws compatible with regional and cultural beliefs of Iranian people. Unfortunately, available laws could not keep up with the advances.

**Conclusion:**

Iran juridical system should conduct and restrict actions in the area of stem cells technology by gathering experts of different political, science, medicine, social and mindful who are familiar with law, according to notions of intellectual jurists and legislators, Islam and Shia religious.

## Introduction

Today, stem cells technology is considered a new approach in treatment of diseases and it has brought hope for production and recovery of body tissues and organs by tissue engineering and regenerative medicine. The world’s great tendency for this technology is because it is promising approach for treatment of diseases. It is predicted that the countries which have this technology are going to be internationally rated as powerful countries. In parallel with the development of this technology, many ethical and religious issues have been raised. Scientific developments, usually encounter moral and religious issues. The research involving embryo stem cells has similar matters in question. Performing experiments on human embryo is still a matter of debate. Since the status of jurisprudence in Iran in the field of stem cells is not obvious and unfortunately the existing regulations could not go along with these developments, the development of legal and institutional infrastructures is considered as the main priority. In order to develop this technology, it is necessary to review the rules of the leading countries in the field of cell therapy technology to draft new laws (1). In this regard, although Shia jurisprudence does not ban these studies, jurisprudential views must be taken serious in this regard (2). Although manipulation of human embryo is a matter of discussion from Shia jurisprudence point of view. In this paper we discussed ethical, legal and religious issues about stem cell research and usage along with pointing to the research on human embryos according to Shia jurisprudence. 

## Materials and Methods

This analytical-descriptive research gathered the relevant data through a literature search. In this study, we described the relationship between the human being and his own stem cells based on the Islamic point on permission and reverence of stem cells technology. We have used PubMed, ScienceDirect, Google Scholar, Iranian databases like SID and Iran doc and also Iranian law and Islamic resources for this review.

### Stem cells

Stem cells are undifferentiated cells that have the ability of proliferation and differentiation into other cells of the body and can be obtained from four sources: i. Human embryo, ii. Adult tissues, iii. Cord blood and iv. Induced pluri-potential stem cells (IPS) which are produced from skin cells (fibroblasts) and adult. In 1998, scientists successfully extracted these cells from the human spare embryos remaining unused from in vitro fertilization (IVF). Adult stem cells can be found in many specialized tissues including the brain, bone marrow, liver, skin, digestive system cord blood can be obtained from umbilical cord of new born and IPSs are produced by inserting genes in fibroblast cells (3). But as these cells have different potencies of proliferation and differentiation, some of them may not exhibit the expected performance. Embryonic stem cells have the highest power among them that cannot be ignored.

Stem cells are expected to be used for repairing the damaged cells of heart, repairing the bone tissues, treatment of nerve diseases and lesions, repairing burn and skin lesions, improving the pancreas and insulin secretion, testing the effectiveness of new medicines, practicing transplantation, producing sperm and ovum, etc… (4).

### Stem cells research in Islamic societies

According to Islam, scientists are not only responsible for medical research for treatment of untreatable diseases, but they are also responsible for proving the benefits of research on embryo for treatment of diseases. Thus, related documents proving that justifies researches on stem cells according to corresponds with moral- religious and functional theory in Islamic world, should be provided. So, Islamic jurists should evaluate the advantages and disadvantages of stem cell research by considering Islamic values in relation with the importance of moral status of human embryo. There are some debates among religious scholars whether embryo possesses personhood and moral and legal dignity or not. According to moral-divine considerations of the catholic religion, the developmental stages of embryo’s growth pass till reaching the personality; in this regard transmigration of the soul and shaping the personality of the embryo happen just after fertilization (5). 

Criminal justice system determines the blood money (*DIAA*) of the embryo based on the age of it and the cause of abortion. Under some conditions, law considers the embryo as a part of the mother’s body (please refer to therapeutic abortion law). In the field of IVF technology, scholars spiritually discriminate between an implanted embryo and supernumerary embryos. In this regard, an implanted embryo has the life rights but the supernumerary embryo is regarded as an aborted embryo, because it is out of the womb and transmigration of the soul did not occur. Thus, there is no punishment for discarding of this kind of embryo before being placed in womb, using these embryos for stem cell researches is allowed because they are potential sources of treatment (6). 

Islamic jurists don’t permit all aspects of using supernumerary embryo because in such permission it seems to treat human beings as a product. Human embryo contains potential life power and it deserves respect but not dignity from the beginning. If the previous statement was not right, why should Islamic criminal laws assign penalty for intentional abortion in its early stages of development but after implantation? The Quran description of the development of embryo till ensoulment and reaching human dignity is an emphasis on the gradualness of establishment of human personhood in human embryo (6). 

This mysterious issue in Islam is shown by terms used for different stages of embryo development from the fertilized egg, blastocyst, and embryo in the womb till birth that are exactly noted in various religious texts. These texts also discuss ensoulment. Checking the majority of religious terms used for “abortion” shows related spiritual and legal dignity issues with more details. In Shia rules, similar to catholic rules, abortion of the embryo is a sin, but in contrary to Catholics’ beliefs, some Shia jurists believe that human life begins at the implantation time. Some people based on the Holly prophet’s statements, believe that ensoulment happens at the end of the 4 months (i.e. 120 days) of pregnancy. 

So it can be said that the Quran’s silence about the embryo’s dignity has given the jurists the possibility to distinguish between the bio and spiritual personality. Definition of levels of the embryo development, at least in the first 3 months of pregnancy, Jurists consider the punishment for abortion of implanted embryos but not for laboratory embryos in the IVF clinics and allow the use of the supernumerary embryos for stem cells researches. Also, as beliefs approve the possibility of the embryo’s life in the last stages of embryo development and since there is no discussion over the development of spiritual capacity of the embryo at first stages, the Islamic jurists require moral justification about using embryos for extracting stem cells (6). Therefore, according to Islam and some scholars, study and research on the embryo for therapeutic purposes can be accepted if done before ensoulment. 

### Stem cells research in Christianity

Roman and Orthodox Catholics believe that there is a connection between humanity and fertilization and development continues till human being reach the stage at which he could physically and spiritually have a picture of God, so all stages of development must be regarded as "HOLY". The Roman Catholic Church opposes the use of fetus in research and treatment. According to the Catholics, human comes to life from the moment of fertilization. Therefore, the human embryo is considered as a complete human and has the right to live, and all embryos must have a chance of development to a complete human and be born. Therefore, it is unacceptable to deliberately eliminate an embryo, even for medical purposes. The IVF process, which normally generates surplus embryos with the potency for becoming humans and making them a fetal source for research, is not a legitimate procedure in the view of Catholic Church. Protestants believe in pluralism and they do not have single source for making decisions and releasing the divine commands to be referred by Christians. Protestant beliefs about stem cells should be judged by the conscience of each individual. According to them, different people can have different ideas on a common topic, and these beliefs must be consistent with Christian beliefs. Some protestant branches believe that a perfect human is gradually created; so, this person may not exist in that primary cell mass and some others believe that ensoulment happens after day 14 of gestational age (7). 

### Stem cells researches in Judaism

Based on the Bible and the Jewish legal rules (Talmud), the human identity does not establish at the moment of fertilization, but during the growth in the mother’s womb. The fetus is a part of mother’s body as long as it is inside the mother’s body. Also, at the birth, the fetus is regarded as a complete human being similar to his mother. Before the first forty days, even as long as the fetus is in the womb, it does not have the moral and legal status of a person, because according to the Jews, the fetus remains as water until the forty days after fertilization (7). 

### The type of relationship between the human being and his own stem cells based on Islamic point of view

At the first glance, the relationship between human being and his own tissues and stem cells was not exclusively discussed in juridical texts alone but in attachments to this topic, organ transplants have been discussed. Since tissues and adult stem cells are found in the organs, the religious view about the relationship between man and his body, is applicable for tissues and stem cells (8).

Stem cells transplantation, is a new phenomenon; so, in juridical texts and traditional laws, it has not been discussed; but, contemporary scholars have discussed and many of them accept organ transplantation from living donor, cadaver and brain dead. Using body organs for organs transplants has two ways: auto graft which is transplantation of humans own tissue to himself or heterograft which is grafting the tissue from a person to someone. From jurists’ point of view, transplantation is allowed following receiving donor’s consent and in the case of living donor permitted if complications of organs transplantation are compensated (9).

To answer the question that whether a man is the owner of his body and he can sell his body organs, tissues, and cells, the Muslim jurists have discussed and it was concluded that human body does not have a price thus it cannot be sold. If the part of the human body is sold as goods, in fact the dignity of human has been compromised (10) based on the holy Quran (11). In this respect, selling human parts and sale contract is not allowed. Unlike paying a price for human part to the donor which is banned, expenses of organ transplantation process including the physician, hospital, etc. can be paid with no problem but just for organ donor who is volunteer of this process, receiving money is banned. Late supreme leader of Iran, Emam Khomeini (peace be upon him) regarded the person as the owner of his body but the ownership was different from ownership of properties. However, from this difference he does not conclude that the human cannot decide for his body, but he believes that a person can decide for his body for treatment as he wishes except for the cases in which there is a legal or religious prevention (12). 

It is obvious that the relationship between the man and organs or tissues of the body cannot be in the form of benefit rights (13) because the profit right is for the lands and properties, thus the profit right cannot be used for body organs. Also, according to the definition of profit right (14), it cannot be applied for the relationship between the man and his organs because for proving this, one must be able to transfer the right to use of his organs to another person by a contract (usufruct right) so, recipient person would be the owner based on transfer the usufruct right to him, in fact, everyone from the birth is the natural owner of his body organs and this kind of relationship indicates that nobody else can be the owner of another person’s body organs (13). Concerning the relationship between the man and his body organs and cells, there are three ideas in Islam that whether the man is able to donate his tissue, cells, and organs to somebody else or has the permission to sell/buy tissues, cells, and organs. 

### The relationship of domination and capture

Some jurists indicated that the human has absolute power over his body (monarchy) (13, 15). In this way, "monarchy" can be used, like people’s absolute power on their body which is a logical phenomenon. Like the way that human has power over his properties, he also has power over his self from any aspect, but based on this view point, organ donation can be permitted (16). Also, the Islamic rule that says: the people are predominant on their properties and lives (17) as a juridical rule describes the human "monarchy" on his body. According to the monarchy and the effects it has, the term natural ownership is better for this relationship (8). 

### Trust relationship

Some jurists believe humans are the God’s properties trustee and they believe human lacks any ownership of his body organs and they believe he does not have any ownership even benefits or shared ownership (18). Based on the above idea, humans are protectors of their body and soul which is against the human freedom authority. Additionally, we are allowed to seize body organs to the extent that the religion permits while the trustee cannot seize but for protection. 

### Ownership relationship

But most of the jurists have called the relationship between the human and his body organs an "ownership relationship". Based on this idea, some jurists believe that in punishment amputation (for example in nemesis or theft), the amputated organ belongs to the person whose organ has been amputated (19). Overall, from the juridical view, human ownership of his body organs is a matter of controversy; these debates are expressed and explained below: 

#### Formative ownership relationship 

Some jurists believe that the existence of something depends directly upon the existence of human beings; in this regard, if we do not exist they could not exist either, they are not separate from us, and so they are our properties. Like body organs and five senses that because of the formative ownership that we have on our body, we are the real owners of them (20). But it seems that we cannot accept this kind of relationship between the human and his body organs because human does not have this kind of complete ownership and authority on his body, while human in religious and juridical sources is banned from harming his body (16).

#### The relation of credit possession

The credit possession established by a clergy jurist or common law is determined for the person who is credited and its area is also determined. Therefore, the source of this kind of possession is wisdom and common law given by the jurist’s signature. Due to the person possession on his/her own body organs is a rational thing that is discovered through the intellectuals unanimous and their practical collusion, and it is not rejected or is defined by the jurist who is the one of the intellectuals, so this kind of possession agrees the religious law (21). Furthermore, unlike the previous statements that says the body organs had no fiscal value, today, common law considers a great value for it and according to the common law, a kind of possession is credited for it. Of course, this problem is acceptable by the intellectual just in special situation that it does not involve some of harmful possessions, so this credit possession lacks free will and unconditional authority which primarily is one of the features of the right possession. 

At the same time, the credit possession is the result of state and forging. So, a forging should be done by a jurist or it is created according to the rational basis when there is no prevention by the jurist. While it cannot be said that this kind of ownership is absolutely accepted by intellectuals, although the intellectuals preferred donation of organ to another person and they accepted saving the life even payment as compensation, from the point of view of intellectuals, these are not important reasons for accepting the personal ownership of body organs like properties; also, it can be the only reason for the personal dominance over her/his body organs (16). 

Concerning credit possession of people on their body organs, it can be said that: first, the source of this it, is not issued by clergy scholars but is the rational deduction, and in religious contexts this rational basis is not improved yet or at least it is uncertain; second, it is assumed that acceptance of this idea faces a lot of limitations that turns its situation into "dominance right and possession" (8). 

#### The relation of innate possession

Personal ownership of organs can be defined as "the relation, dominance or any permanent right that arranged between mankind and his/her organs and the legislator validated it and with such right, the person can have authority of his/her own organs legally, in a way that they can benefit from all possible profits and no one can prevent them" (13). Most religious scholars approve the idea that the human an original ownership of his own organs (21, 22).

Intuitional and rational approaches also approve such kind of ownership and authority, as the religious scholars and law. Many phrases are common in the language of religious scholars and law that validate the presented relation and significance discussions including "protection of the human body integrity" that indicate that the legislator respect such dominance and mastery (8). 

### The religious view on permission of stem cells technology 

#### Legit rule and the legitimate law 

Legit rule " *ESALAT al EBAHA*" means everything is acceptable and lawful unless there is a clear order against it in Islamic texts. In case there is no reason for the permission or prohibition, the law considers it "permissible" (23). The law or legitimate rule is also equipoised as the legit rule. Based on this rule, human has a right of any involvement and occupation of things nearby (24). 

Legit rule and the legitimate law are the bases for clergy scholars to permit new topics of life technology. 

### The human ownership of their organs and cells

A) With reference to the prophetic tradition "people have dominance over their body and property" (25). This rule shows the authority of people over their own properties and body; thus, people must legally have this authority different organs of their body. 

B) Sacrifice is praised: "is there anyone who sells his life just for God sake? (it resembles Imam Ali (peace be upon him) who slept in prophet’s bed in the night of prophet’s migration to Madina), and God is compassionate and kind with his servants" (26). This verse indicates that God gives the option of personhood to human and it is for this reason that he takes action of sacrificing for a good reason like altruism (27).

According to the first rule, people rightfully own their body organs and cells, so they have the right to sacrifice it. Based on this view, there is no difference between sacrificing and donation of an organ or cells for saving the life of a Muslim (22). Concerning this argument, it should be said that it is correct according to dominancelaw that everyone has the complete authority on oneself and owns properties and they are free; however, the holly religion Islam has defined some limit and boundaries for this freedom that must have benefit and no harm from this freedom, it should be within religious rules framework and does not interfere with others rights. The ownership of body is limited to the any harm to wealth. Although, the person will be recognized with dizziness or dementia or any situation that decreases the capacity of the person for making decision, this person will be banned from any ownership to the body. Furthermore, human authority on himself is limited up to wasting his life or making harm to an organ and no one is allowed to make harm to himself, but some harms can be tolerated and can help others to live better for example blood donation or kidney donation. In such cases, this can be considered as sacrifice and is accepted (27).

C) Another proof that can be noted for the person’s authority over himself in Islam, is the ability of the person to converts nemesis punishment to blood money. 

Thus, altruism is appreciated and advised in Islam mostly in properties like food and drink. However, the "altruism" can be increased to the level of sacrifice for the Muslims. Therefore, a Muslim can donate an organ of his body for transplantation for saving another Muslim’s life.

Together, if the reason of transplantation of an organ, tissue and cell is that these are person’s properties, after cutting them off, they are still his properties and he can sell it as he likes; but, selling an organ is not acceptable if we believe that organs and cells are not one’s properties, but he has the right to use them, and donate them for altruistic reasons. 

### The principle of innocence

The most Shia jurists believe that whenever after gathering justifications and juridical documents, there is still doubt in some actions, that action cannot be acceptable and should be avoided. Of course, when there are no valid reasons for religious scholars on permission or banning the usage of stem cells technologies, they approve it. It means that, although the jurist may not find a reason from the real juridical law and Islamic texts, due to necessity of the topic based on scientific documents, the statement is given in the absence of an existent cause for forbidding it, it becomes permissible (26). 

### The juridical view on accepting the stem cells technology 

#### Changing the divine creation

Mainly, the jurists of Sunni point to changing the creation of GOD with the reference to Holy Quran Sura Nesa (4^th^) verses 117-119, which reminded that the devil will misguide people and instruct them to change God creations: "instruct them to change people" (28). In addition to this verse, in another source, it is said that: "do not change the people" (29). From both of these two verses, the changing God’s creations is forbidden (*HARAM*) (24). By this argument, some Sunni clergy scholars state that many new technologies can be obvious proofs for changing the God’s creation, so, it is forbidden (30). 

In response to this argument, it can be said that first, the meaning of changing creation in the notable verse is shifting and distortion in the right religion and God’s world not the absolute changing in creatures (31); second, the belief in this argument in general, prevents many normal actions that are allowed in Islam religion (32). So, there is no benefit in this argument for justification of biological or medical issues. 

#### Human does not own his organs

Legislators believe that a person is not the absolute owner of his body and this body is just a god-given gift and every ownership in this gift needs the owner’s permission and the person has the authority to use body parts except in some special cases. This reason is disputable from different aspects; first, the purpose of the absence of man ownership on his own body is not explicitly clear. If the meaning of this reason is to prevent commercial organ donation, it would be acceptable; but this meaning cannot be a respect for body parts; second, does "deposit and safekeeping of the body", have the same meaning as authority? If it is so, the body of no one is not a deposit for them. The last word is that the ownership of the body does not prohibit the safekeeping (24). 

#### Argument of "No harm"

The rule of "no harm in Islam" declares that, there is no harmful law in Islam meaning that Islamic statements have only benefits. The harm is defined as "a defect in property and life" and the meaning of loss is “exacerbation, restriction, severity and difficulty". This argument is along with both ownership and gift argument and a person must not harm his body even if he is absolute owner of it (27). 

### Precaution rule 

Precaution rule maybe is the reason for banning stem cell technology; it is justifiable that other reasons and religious proofs relevant to this issue, are not sufficient for this purpose. Precaution rule, also known as (practical rule), is the statement of the jurist or legislature about the necessity of considering all possible tasks in cases that the task could be trusted and in the source of the task but there are doubts about its application in the life (23). On this basis, a scholar should act in a way that he was sure about application of the task along with the source of it. This is opposite of legit rule that says the task is OK when there is nothing absolutely against it. 

## Discussion

The jurists by considering process of embryo growth and evolution, at least in the first three-month of pregnancy, study the rules that deny the personhood of "supernumerary" embryo before implantation in I.V.F clinics and they allowed using the unneeded embryos in stem cells research (5). 

From Islamic jurisprudence point of view, there are three theories for the relationship between man and his organs and body cells: dominance and ownership relation, safekeeping and ownership; according to the meaning of dominion, it is clear that between the meaning of dominion and the meaning that is presented for the ownership, there is not a difference from the nature aspect, so it seems that using the ownership word is better for this relationship, because dominion is a result or a statement of possession commands (8). On the basis of ownership theory, also people are just changed to conservators of their own soul and body and this order is against the freedom and person authority. Furthermore, we are allowed to take authority of our body parts in juridical limitations while a trustee does not have the ownership right, except for protection. Most jurists that expressed their opinion in this area regard the human relationship and his body as a kind of owning relation (19) that briefly includes originating ownership, credit and innate; according to the originating ownership while God is the owner of all the world, we also have a kind of originating ownership about organs and parts of body. But, the man does not have total authorization over his body; also, based on religious and juridical texts, causing injury and harm to the body is prohibited (16). About the credit possession of the person on body organ, it can be said that first, the basis and source of it is thinking because that does not have rational basis about the ownership on body organs is not proved or it is in doubt; second supposing that it is accepted, this kind of ownership faces many limitations making it "dominance and possession" (8). But, most of jurists agreed innate authority of human being on his own body. Intuitional approach and rational basis confirmed this kind of ownership and dominance, as the jurists and scholars confirmed it (8). 

Most of Shia jurists believe in permission and legit. The main reference reason toward recognizing a task statement for applying these technologies is "legit rule" and the "legitimate law". Referring to these rules beside rejecting of forbidden reasons and responding to them, make permission and legit application for these technologies. The statement that relies on the "legitimate law" and "legit rule" in new topics in biomedical and biotechnological fields, are the first reasoning of Shia scholars leading to the permission. Regarding the person ownership over his body parts and cells, it can be said that being praised of sacrifice cannot be the reason of donation of cells or organs. Importantly, in typical affairs, the person has a right to prefer others right to oneself. So, a Muslim can separate a part of his body and gives it to save another Muslim’s life. As a result, if the reason for justification of transplanting an organ, tissue and cell is that those body parts are donor’s properties, after removing them, they are still his properties, and he can sell it in any way he likes; but if we don’t consider body parts as properties, and he has only the right of using it, there is no right for selling body parts for money (31). 

Reasons indicating the absence of permission and reverence of this technology include the changing reverence in God creation, the absence of human possession on his organs, referring to the profitable and harmless law and following the precaution rule. In response to the argument of change in divine creation, it was said that change in creation has been meant distortion in the divine religion and nature, and only it has been considered as absolute change (31). Second promise to this word in general and total is in conflict by many permission of actions that are allowed in Islamic religion (32). As a result, it cannot be used for confirming reverence and prohibition in this topic. Permission in taking authority is enough, even if it issued by the real owner. The evidence for this is that human being can naturally and continually make several changes in his body and organs by jurists’ permission and none of these kinds of possession are illegal (24). Reasoning to the usage of stem cells technologies makes it acceptable, when it is performed carefully and safely. These technologies in special situations, maybe are harmful and dangerous for people life and society. The topic is a matter of discussion that even though from the point of view of others, do not contain in jurists tasks; however, seeking the religious statement relevant to the topic will be different by multidirectional cognition of that topic even by using the external knowledge of jurists. To sum up, causing harm to the body, is illegal and forbidden according to the scholars’ point of view. Besides, statements about the need for prevention of harm in some juridical subdivisions are also referred by Shia juridical. Since it should be considered that the stem cells technologies maybe are not safe to use for human, can lead to prevent harm argument. Also, resort to precaution rule, it is advisable to calculate risk and benefit and consider safety of using these technologies before approving them although this consideration is not religious (23). 

## Conclusion

It can be concluded that as currently there are so many controversies about safety of these cells and possible dangers for human body, till getting new advances and clearness of risks and benefits, also discovering prevention ways with these dangers and securing the usage of these technologies, we should follow precaution rule. 

As delay in accepting the stem cells technologies deprive human from potential treatments for incurable diseases, Islamic scholars and governments should assess risks and benefits of stem cells researches to approve them. On the basis of Islamic values related to the moral status of embryo, and according to the superior moral, cultural and religious values of the Islamic society of Iran accepted the action towards development of these researches. Iran juridical system should, according to notions of intellectual jurists and legislators, Islam and Shia religious, conduct and restrict actions in the area of stem cells technology by gathering experts of different political, science, medicine, social and mindful who are familiar with law, philosophy and divinity, also express the existence challenges and exit methods, and try to pass codes and design guidelines to fill legal and ethical gaps. 
